# Avulsive Achilles Tendon Rupture in a Patient With Alkaptonuria: A Case Report

**DOI:** 10.7759/cureus.62631

**Published:** 2024-06-18

**Authors:** Luís Fabião, Guilherme França, Anita Cunha, Bruno S. Pereira, Nuno Esteves

**Affiliations:** 1 Orthopedics and Traumatology, Unidade Local de Saúde de Barcelos/Esposende, Barcelos, PRT; 2 Orthopedics and Traumatology, Unidade Local de Saúde de Braga, Braga, PRT; 3 Orthopedics and Traumatology, Clínica Espregueira - Fédération Internationale de Football Association (FIFA) Medical Centre of Excellence, Porto, PRT; 4 Orthopedics and Traumatology, Trofa Saúde Braga Sul, Braga, PRT; 5 Rheumatology, Unidade Local de Saúde do Alto Minho, Ponte de Lima, PRT; 6 Orthopedics and Traumatology, Hospital Lusíadas Braga, Braga, PRT; 7 Orthopedics and Traumatology, Dom Henrique Research Centre, Porto, PRT; 8 Orthopedics and Traumatology, School of Medicine, University of Minho, Braga, PRT; 9 Foot and Ankle Unit, Human Anatomy Unit, School of Medicine, University of Barcelona, Barcelona, ESP; 10 Orthopedics and Traumatology, Hospital CUF Trindade, Porto, PRT

**Keywords:** homogentisic acid, ochronosis, alkaptonuria, rupture, achilles tendon

## Abstract

Alkaptonuria is a rare autosomal recessive disease caused by a mutation in the homogentisate 1,2-dioxygenase (HGO) gene, leading to the accumulation of homogentisic acid (HGA). HGA polymerizes to form a black pigment that accumulates in connective tissue and joints (ochronosis), causing their destruction. In this work, we report a case of Achilles tendon rupture in a patient with a prior diagnosis of alkaptonuria.

A 71-year-old man presented to the emergency department reporting pain in his posterior right ankle and dysfunction, evolving over three weeks after falling down a short flight of stairs. He had previously been diagnosed with alkaptonuria and had undergone five joint prostheses and an aortic valve replacement. A physical examination revealed right ankle edema, pain upon palpation, a palpable gap at the insertion of the tendon, and a positive Thompson test. An MRI confirmed an avulsive rupture at the insertion of the Achilles tendon. During surgical exploration, black pigmentation was observed in the tendon, which was reinserted using a double-row system reinforced with a percutaneous Bunnel stitch.

The patient was discharged the following day with a cast splint, maintaining the foot in physiological plantar flexion for two weeks. In the subsequent two weeks, he used a non-weight-bearing walker boot, and finally, in the following two weeks, he began weight-bearing. Two months post-operation, he was walking without support. Twelve months after the intervention, the patient regained their previous functional status, being able to walk on tiptoes without difficulty.

Spontaneous Achilles tendon rupture without associated trauma in patients with ochronosis is rare, with limited literature demonstrating successful outcomes post-surgery. Since the tendon becomes more fragile due to pigment accumulation, it was reinserted using a double-row system, increasing the contact area and more effectively distributing the load.

There is no standard technique for treating these patients, but the patient's previous functional capacity was restored, with no new ruptures to date. The significant morbidity of alkaptonuria and potential complications, such as tendon ruptures, warrant future studies to discover and develop new prophylactic and therapeutic treatments.

## Introduction

Alkaptonuria is a rare autosomal recessive metabolic disorder that is caused by a mutation of the homogentisate 1,2-dioxygenase (HGD) gene, which leads to HGD deficiency, which in turn is part of the phenylalanine and tyrosine degradation processes [1]. With this pathway affected, homogentisic acid (HGA), an intermediate metabolite of phenylalanine and tyrosine metabolism, will accumulate, oxidate to benzoquinone acetate, then polymerize and form a dark pigmentation [2] that accumulates in the connective tissues or the joints [1,3], leading to bluish-black pigmentation, called ochronosis [4]. HGA is excreted in urine, turning the urine dark, which is usually the first sign of the disease [2]. Other signs and symptoms include coloration of the ear cartilage, sclera, bone, cartilage or tendons, arthritis, joint destruction, rupture of the tendons, muscles or ligaments, stone formation (kidney stones and prostate stones), osteopenia, and osteoporosis [1,3].

Alkaptonuria was the first disease accepted to follow the classic Mendelian recessive inheritance, and Archibald Garrod was the physician who first described this condition in 1908 [5]. Its estimated prevalence is 1:250000 [6]. In this paper, we will report a case of Achilles rupture in a patient with a previous diagnosis of alkaptonuria. This is a rare complication that was first described in 2003 [7]. There are few cases reported of this complication in the literature. The aim of this report is to alert other physicians to these complications, which can lead to better prevention and delay of such complications.

This article was previously presented as an e-poster at the 41º Congresso Nacional de Ortopedia e Traumatologia on November 7-9, 2022, and at the 60 Congreso Nacional de la Sociedad Española de Cirugía Ortopédica y Traumatología on September 27-29, 2023.

## Case presentation

A 71-year-old male patient presented to the emergency department with complaints of pain in the posterior region of the right ankle and an inability to walk. These complaints evolved over three weeks after a fall from a short set of stairs.

**Figure 1:**
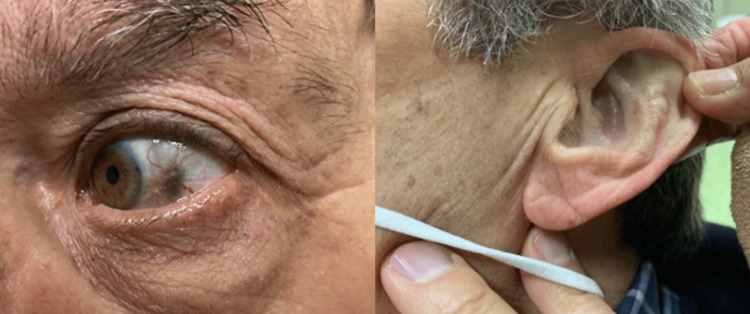
Ochronotic pigmentation of the sclera and ear cartilage

The patient's medical history was remarkable for known alkaptonuria. Worth noticing in his surgical history are bilateral total hip prostheses, bilateral total knee prostheses, total left shoulder prostheses, and aortic valvuloplasty. In all these previous surgeries, ochronosis was described (Figure 1).

On physical examination, the patient had ankle swelling with pain and tenderness on palpation. There was a palpable gap in the Achilles tendon at the level of its insertion and a positive Thompson squeeze test.

**Figure 2:**
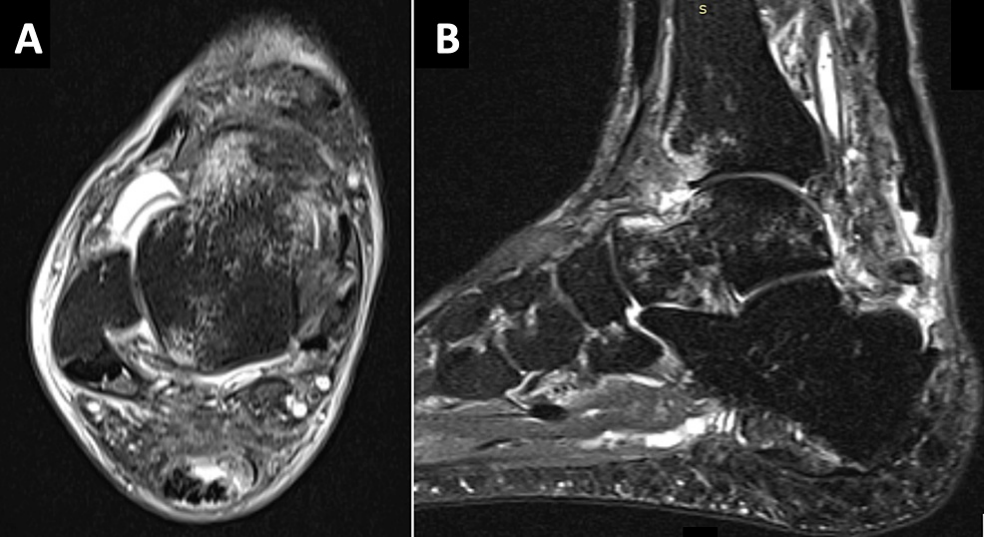
MRI showing an insertion rupture of the Achilles tendon and a rupture of the FHL: (A) T2 MRI axial images, (B) T2 MRI sagittal images FHL: flexor hallucis longus, MRI: magnetic resonance imaging

An MRI was ordered, demonstrating a complete avulsive rupture of the Achilles tendon insertion and a rupture of the flexor hallucis longus (FHL), probably chronic (Figure 2).

**Figure 3:**
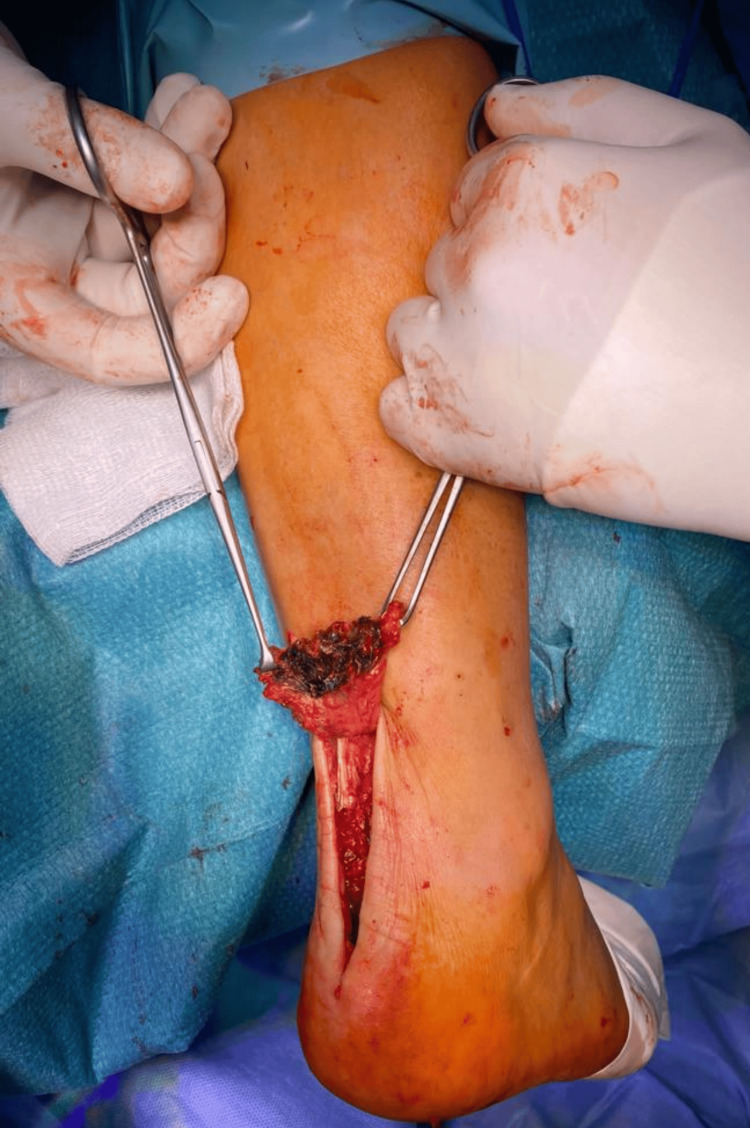
Surgical exploration showing a complete right Achilles tendon rupture at its calcaneal attachment with dark-black pigmentation of the frayed end

It was proposed for surgical repair, which the patient accepted. During the surgical exploration, a complete rupture was observed in the calcaneal insertion of the Achilles tendon, and a black pigmentation was found in the tendon (Figure 3).

The tendon was reinserted in a double-row configuration, augmented with a percutaneous Bunnel stitch, without complications. Two anchors were attached to the posterior tuberosity of the calcaneus, with one suture tape and one suture thread each. The four ends of the suture tape were passed through the tendon and attached to two knotless anchors in the distal row. The proximal anchor suture threads were then used to perform augmentation with a Bunnel stitch, extended proximally in a percutaneous fashion to improve the purchase of the healthy tendon.

**Figure 4:**
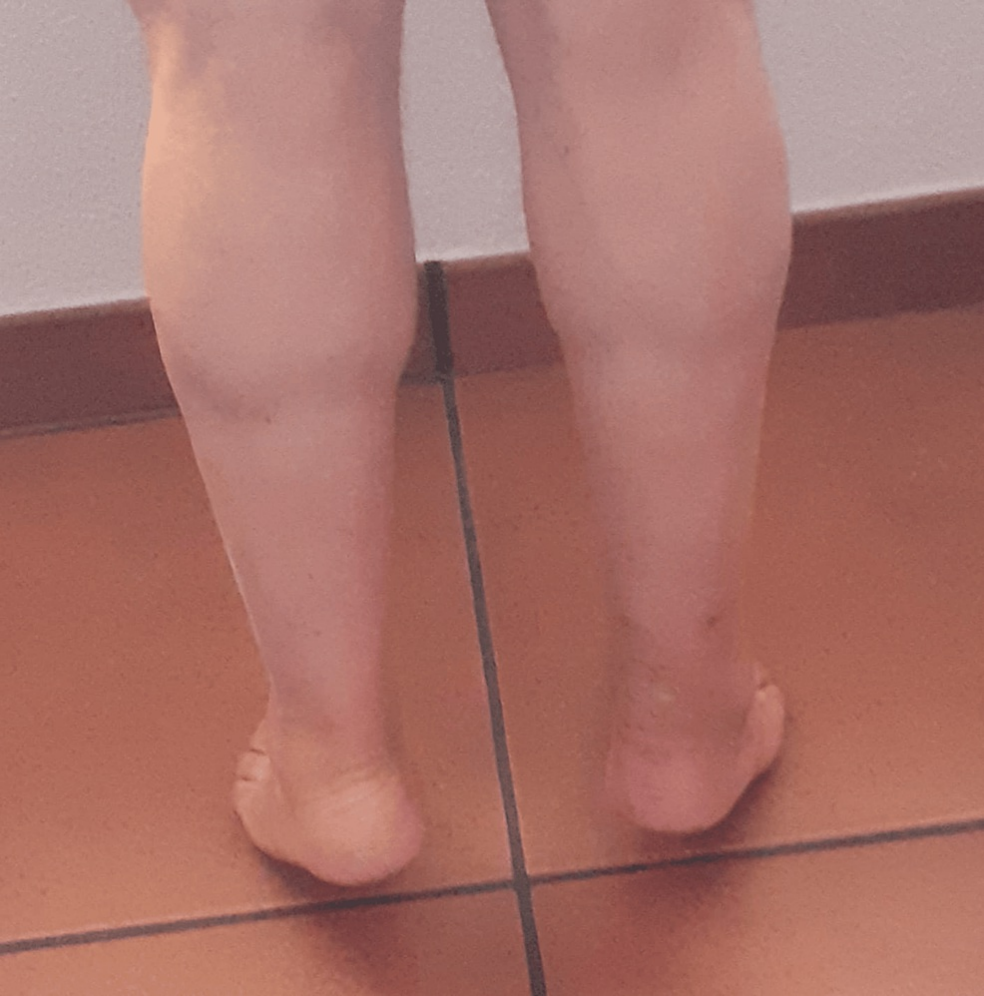
At 12 months of follow-up, the patient is able to bear weight on the forefoot without difficulty

The patient was discharged from the hospital the following day after the surgery with a posterior splint with the foot in gravity equinus. Two weeks later, the cast was replaced by a moon boot with calcaneal edges and removed progressively in the following two weeks. At six weeks post-op, the patient removed the moon boot and started physiotherapy. Twelve months later, he came to the hospital walking on his own, without any external walking aids (Figure 4).

## Discussion

Alkaptonuria patients are usually asymptomatic up to the fourth decade of life, and at this time, the manifestations of ochronotic osteoarthropathy begin to appear [2,6]. HGA accumulates in the connective tissue, causing irreversible damage to collagen [8]. Tendons have a large amount of collagen in their structure; thus, disease progression increases the probability of tendon rupture [1]. There are no specific findings for the diagnosis of ochronosis in tendons. Spontaneous Achilles ruptures without any associated trauma are rare, and there are few published cases demonstrating success after surgery.

Since the tendon is more fragile due to the accumulation of the pigment, we reinserted it with a double-row system, as it increases the contact area and distributes the load more efficiently. Slower progress through the rehabilitation stages was ensured due to the increased risk of recurrence. In some situations, an augmentation with FHL is chosen [6]; however, in this patient, it was not possible since the FHL was chronically worn out. In cases of an increased gap, the procedure is not yet well defined. However, Mwafi et al. [6] proposed the following: if the patient has a gap of 1-2 cm, end-to-end repair with or without tenodesis augmentation is usually performed; if the gap is 2-5 cm, V-Y advancement with or without tenodesis augmentation is typically used; and finally, if the gap is greater than 5 cm, autograft or allograft tendon transfer or reconstruction is recommended. In this article, they also report that the FHL is the best tendon for reconstruction of the degenerated tendon for all defects greater than 2 cm.

There is no effective treatment for alkaptonuria. The aim is to prevent and reduce damage, and some treatment options are described. Some studies show that vitamin C intake may slow down HGA polymerization. Also, diet changes such as the restriction of foods containing phenylamine and tyrosine can limit symptoms due to the reduction of HGA synthesis. Physiotherapy and anti-inflammatory drugs can relieve symptoms but do not change the prognosis of the disease [8,9]. Nitisinone is a potential treatment that can modify the prognosis by inhibiting the formation of HGA [6,9]. When large joints are involved, glucosamine and chondroitin are reported to reduce joint complaints [10].

## Conclusions

This was a challenging case. No standard technique has been previously described to treat these patients, but we managed to get the patient to recover to his previous functional capacity without further rupture in the following three years. Alkaptonuria's significant morbidity and potential complications like tendon ruptures give grounds for future studies to discover and develop new prophylactic and therapeutic treatments.
